# Lunacy Law for Medical Men

**Published:** 1895-03

**Authors:** 


*Lunacy Law for Medical Men.* By CHARLES MERCIER, M.B. Pp. iv., 148. London: J. & A. CHURCHILL. 1894.

Dr. Mercier's name is so well known in lunacy that we felt confident that a book dealing with the Lunacy Law from his hands would be valuable. This book, though small, is yet so excellent and systematic in its arrangement, and so lucid in the setting forth of its subject, that no medical man using it as his guide need doubt his ability to understand the Lunacy Act of 1890 so far as is necessary. Every practitioner should provide himself with a copy for his writing table, in order to have a handy compendium by him of the proceedings which are prescribed by law with respect to dealing with insane persons.

Medical men for some time past have been trying to put aside one of their responsible duties which is of the greatest importance to society as well as to their patients. Signing medical certificates for the insane has been avoided under every pretext possible for fear of grave supposed risk attending their so doing. As Dr. Mercier well observes: "Such an attitude on the part of a medical man is hardly more reasonable than that of a patient who refuses food for fear it may contain poison." He also says: "It is scarcely too much to say that if the rules and precautions recommended in this book are followed, the risk is altogether obviated."

